# Prevalence and Factors Associated With the Informal Market for Medicines in Benin: A Community‐Based Cross‐Sectional Study

**DOI:** 10.1002/hsr2.71170

**Published:** 2025-08-19

**Authors:** A. Narcisse D. Agbanou, Fernand A. Gbaguidi, Fidèle Zinsou, Yossounon Chabi, Habib Ganfon

**Affiliations:** ^1^ Benin Agency for Medicines and Health Products Ministry of Health Benin; ^2^ Medicinal and Organic Chemistry Laboratory, School of Pharmacy, Faculty of Health Sciences University of Abomey‐Calavi Cotonou Benin; ^3^ Ministry of Health Benin; ^4^ Monitoring Board of the Pharmaceutical Sector Regulatory Authority for the Health Sector Benin

**Keywords:** Benin, cross‐sectional studies, housing, informally sold medicines, patients' purchasing behavior, pharmacies

## Abstract

**Background and Aims:**

Medicines are intended to improve the health of individuals with various medical conditions, but their poor‐quality poses significant risks, especially in low‐ and middle‐income countries like Benin, where informally sold medicines are widely consumed. This study assessed the prevalence and factors associated with their consumption in Benin.

**Methods:**

Conducted in 2018 across 72 districts of Cotonou, it targeted 864 households. Data collected included the consumption of these medicines, along with sociodemographic and socioeconomic information, as well as medicine‐related practices such as purchasing habits, self‐medication, medical consultation, access to the informal market, awareness of risks, and reasons for consumption.

**Results:**

The results revealed a pattern of multiple‐source medicine procurement, with 25.5% (95% CI: 22.5%–28.5%) of individuals reporting the use of informally sold medicines, despite 83.84% of households obtaining medicines from pharmacies and a health service utilization rate of 58.1%. Factors associated with informal medicine consumption included a lack of awareness of the risks (PR = 2.31; 95% CI: 1.57–3.40; *p* < 0.001), exposure to malaria or pains (PR = 2.02; 95% CI: 1.04–3.94; *p* = 0.04), self‐medication (PR = 95.02; 95% CI: 25.57–353.15; *p* < 0.001), frequent purchases (PR = 1.90; 95% CI: 1.16–3.10; *p* = 0.01), irregular medicine availability in pharmacies (PR = 3.42; 95% CI: 2.26–5.16; *p* < 0.001), and other sources purchases (PR = 15.21; 95% CI: 7.52–30.78; *p* < 0.001).

**Conclusion:**

Informal medicine use remains prevalent in Benin and is significantly driven by lack of awareness, exposure to malaria or pains, self‐medication, frequent purchases, irregular medicine availability, and infrequent pharmacies use. Addressing this issue requires strategic interventions such as raising public awareness and improving availability and access to quality medicines through authorized facilities.

## 1 Introduction

Access to safe and effective medicines is a fundamental component of healthcare and is essential among the targets toward achieving good health and well‐being of populations [1]. However, in many low‐ and middle‐income countries (LMICs), the prevalence of falsified and substandard medicines poses significant threats to health [2, 3]. A particularly concerning manifestation of this issue is the widespread practice of informal pharmaceutical sales, commonly known as “street medicines”, which involve the unregulated distribution of pharmaceuticals outside licensed pharmacies, often in open markets or by vendors [4].

Since at least the 1980's in several African countries, informal pharmaceutical medicines selling is a widespread practice, particularly in the French‐speaking countries [4]. These poor‐quality pharmaceuticals are often distributed through informal markets, where regulation and quality control are minimal or nonexistent [5]. In Africa, inadequate regulation further exacerbates the problem [6], leading to serious health, social, and economic consequences. Africa has the highest prevalence of poor‐quality medicines, with an 18.7% prevalence of falsified and substandard medicines in low‐and middle‐income countries worldwide [7, 8, 9]. Every year in Africa, millions of people die from preventable causes due to falsified and substandard medicines [9]. According to the WHO, most people in the world dying from fraudulent antimalarial medications live in Africa, where it is estimated that 200,000 individuals pass away each year [10].

In Benin, the informal medicine market, commonly referred as “street medicines”, carries a high risk of counterfeit or substandard products [4]. Despite these risks, a significant proportion of the population in Benin relies on these informal sources for their pharmaceutical needs [11]. However, the specific factors associated with the consumption of informally sold medicines in Benin remain poorly understood. Abdoulaye et al. [11] studied informally sold medicines in Cotonou 20 years ago, highlighting affordability, accessibility, and inconsistent availability in the formal health system as key factors. An update is needed to reflect recent changes. In 2018, a more recent study by Apetoh et al. [4] focused specifically on anti‐malarials used in pediatric treatment in Cotonou. This study confirmed high informal medicine consumption among disadvantaged groups but addressed only part of the issue. The time gap and limited scope highlight a need for further research on other medicines and current informal market dynamics.

Benin has a total of 391 pharmacies spread across an area of 114,760 km², serving a population of 13.5 million people [12, 13]. In the Cotonou area, which covers 79 km² [4], there are 112 pharmacies serving a population of 679,012 inhabitants [12, 13]. In Benin, the national regulatory body for pharmaceuticals, which coordinates the national policy on medicines and other health products, has undertaken numerous significant initiatives aimed at combating this threat and improving public health in the country [4, 14]. Since February 2017, an unprecedented repression was organized by the government of the Republic of Benin [4]. This repression led to the seizure of 104 tons in October 2017 and an additional 14 tons later, totalling 118 tons in December 2017 [3, 15]. Despite these efforts, the problem persists due to the global scale of falsified medicine proliferation. A matching range of interventions tailored to the local contexts will be required for the control of informally sold medicines [2].

To combat the sale of these products, it is crucial to identify the factors driving this phenomenon nationwide. This study aimed to determine the prevalence and factors associated with the use of informally sold medicines in Benin. Understanding these factors is vital for devising effective strategies to curb substandard and falsified medicines. The findings emphasize the need for targeted public awareness campaigns and improved access to quality medications through the formal health system.

To investigate these factors, we conducted a community‐based cross‐sectional study in Cotonou using household‐level data. The analysis combines descriptive and inferential approaches to identify key factors influencing the use of informally sold medicines. The remainder of the article is structured as follows: first, we present the characteristics of the study sample; next, we explore the associated factors and patterns of informal medicine consumption; finally, we discuss the implications of the findings and offer context‐specific policy recommendations.

## 2 Methods

### 2.1 Study Design and Study Population

A descriptive and community‐based cross‐sectional study was conducted from January 23, 2018, to February 12, 2018, on 864 selected households in Cotonou. The sampling population was represented by households living in the municipality of Cotonou who met the inclusion criteria. Participants included were fathers, mothers, or heads of households who were at least 18 years old, had resided in Cotonou for at least 6 months before the study, and had provided informed consent.

### 2.2 Sampling Technique and Sample Size

The sampling technique was an equal probability cluster sampling. The sample size was determined by the Schwartz formula:

$$n=\frac{Z_{\alpha }²\mathrm{pq}}{i²}\mathrm{DEFF}$$

Where *n* = 708 represents the sample size, α represents the risk of Type I error (0.05, corresponding to $Z_{\alpha }²$ = 3.84), *p* is the prevalence of informally sold medicines consumption estimated at 35.6% (according to a 2003 study in Cotonou by Abdoulaye et al. [11]), *q* is 1−*p* which equals 0.64, i is the desired precision (0.05), and DEFF is the design effect equals 2, according to Abdoulaye et al. [11].

**Figure 1:**
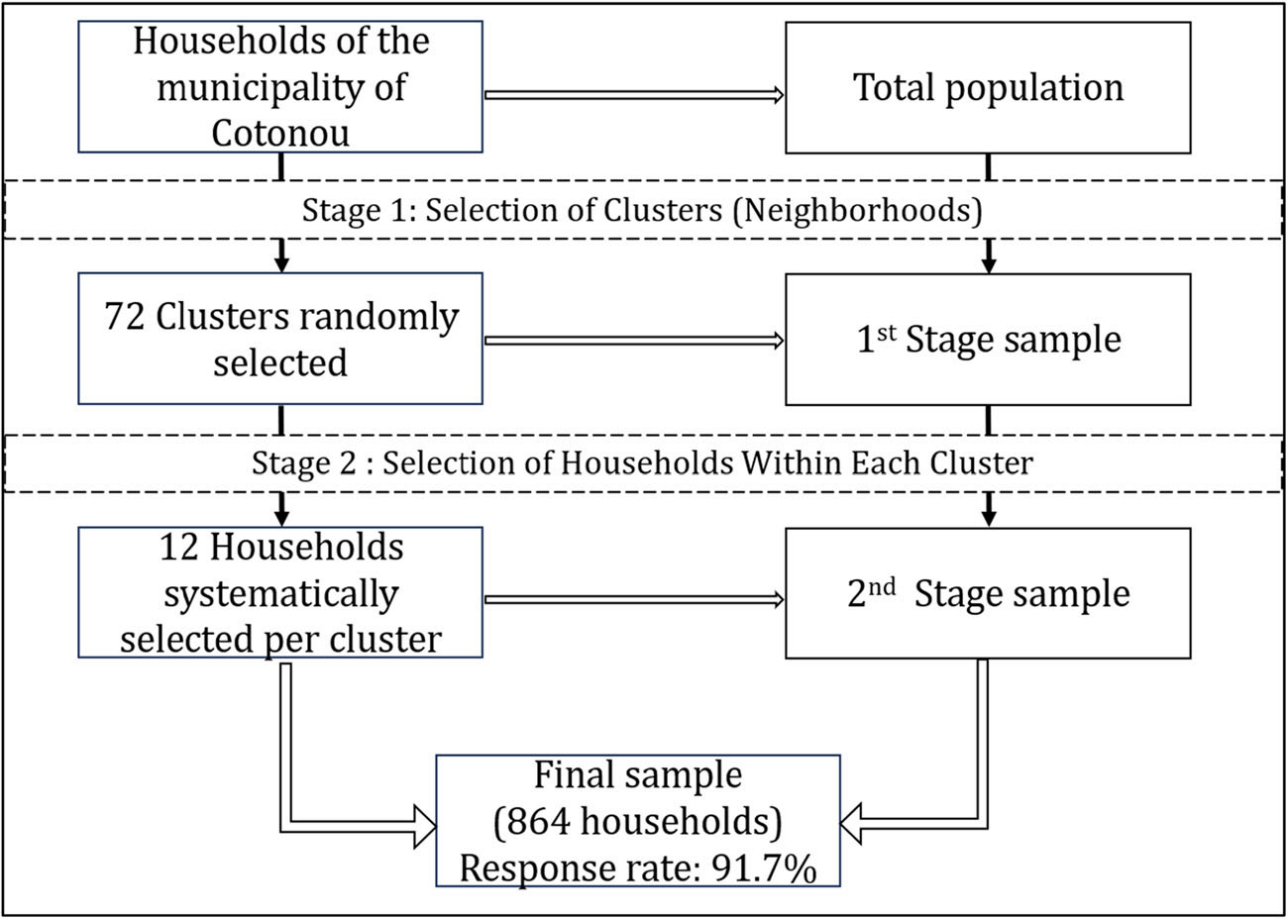
Multiple stage sampling.

Considering non‐respondents rate estimated at 20%, we included 864 households divided into 72 clusters in our study. The size of each cluster was 12 survey units: the survey unit being constituted by the household. Of the 864 eligible households included, 792 agreed to participate, representing a participation rate of 91.7%. The multiple stage sampling is illustrated in Figure 1 below.

### 2.3 Data Collection Procedure

The data was gathered via a questionnaire during an interview in French, local languages (Fon, Goun, Mina, Dendi, etc.) or in English. The questionnaire was administered face‐to‐face to the study participants by a survey team. The survey teams consisted of five (05) pairs, each comprising an epidemiology technician and a pharmacist.

The outcome variable, informally sold medicine consumption, was binary, categorized as “Yes” or “No”. Independent variables were grouped into demographic, socioeconomic, and medicine use‐related categories. Demographic data included age, gender, household size, professional situation, and marital status. Socioeconomic variables covered wealth quintiles and university education levels, with wealth assessed based on housing, audiovisual equipment, means of travel, daily household expenditure, and perceived quality of life. Medicine use data included the number of monthly purchases, purchase locations, main ailments treated with informal medicines, self‐medication practices, medical consultation reflecting health service utilization the last 3 months, adherence to prescriptions, awareness of risks associated with the consumption of informally sold medicines, and pharmacy medicine availability.

### 2.4 Ethical Considerations

The study is secondary analysis of a baseline data published in Médecine Tropicale [11] which was done after ethical approval from the National Ethics Committee for Health Research—Ministry of Health, Benin. In accordance with the pre‐existing protocol, the study was conducted in compliance with ethical considerations and informed consent was obtained from all participants. Each respondent's participation was coded, and data sheets were accessible only to the research team. In addition, the study was conducted in compliance with ethical rules and the Helsinki Accord.

### 2.5 Data Processing

Data entry and statistical analysis were performed using Epi Info version 7.2.1.0. Descriptive statistics were used to provide an overview of the sample characteristics, including the prevalence of informally sold medicines consumption and various socioeconomic, demographic, and medicine use‐related variables.

Sequential logistic regression models were developed to examine factors associated with informally sold medicine consumption. Initially, univariate logistic regression analyses identified factors with a significance level of *p* < 0.20, which were then included in the initial multivariate model. The likelihood ratio test was used to evaluate statistical significance.

Subsequently, we performed a stepwise backward elimination of variables included in the initial model, starting with the variable that had the highest *p* value. Only variables with lowest *p* values and a significant association at the 5% significance level, according to the Wald test statistic, were retained in the final model.

We used prevalence ratios (PR) with their 95% confidence intervals (95% CI) to estimate the strength of the association between informally sold medicine consumption and the independent variables. We concluded a risk factor if PR > 1 with a 95% CI not including 1, and a protective factor if PR < 1 with a 95% CI not including 1. If PR = 1, then the variables were considered independent.

## 3 Results

### 3.1 Background Information of the Respondents

The demographic and socioeconomic characteristics of the participants are summarized in Table 1.

**Table 1:** Demographic and socioeconomic characteristics of the respondents.

Variable	Count (792)	Frequency (%)
Age		
Minimum, maximum	18, 88	—
Median (Q1, Q3)	38 (29, 48.5)	—
Mean (standard deviation)	39.90 (13.70)	—
Gender		
Male	330	41.7
Female	462	58.3
Marital status		
Single	105	13.2
Married	612	77.3
Divorced	15	1.9
Widowed	60	7.6
Educational attainment		
Informal	191	24.1
Primary	182	23
Secondary	269	34
Tertiary	150	18.9
Wealth quintiles		
Poor	372	47
Average	187	23.6
Very poor	148	18.7
Rich	67	8.4
Very rich	18	2.3
Professional situations		
Self‐employed	479	60.5
Employee (civil servant or private)	173	21.8
Unemployed	140	17.7

*Note:* Q1 = First quartile; Q3 = Third quartile.

The average age of the respondents was 39.90 ± 13.70 years (range: 18 years and 88 years). Approximately 58.3% of the respondents were female, and 77.3% were married. In terms of educational attainment, most respondents had secondary education (34%). About 24.1% of respondents reported having received informal education, 23% had primary education, and 18.9% had tertiary level education. Nearly half of the respondents were classified as poor (47%). 23.6% were average, 18.7% were very poor, while 8.4% were rich, and 2.3% were very rich. Regarding the professional situation, the largest group consisted of self‐employed (60.5%). 21.8% were civil servants or private sector employees, and 17.7% were unemployed.

### 3.2 Prevalence and Patterns of Informally Sold Medicines Consumption

**Figure 2:**
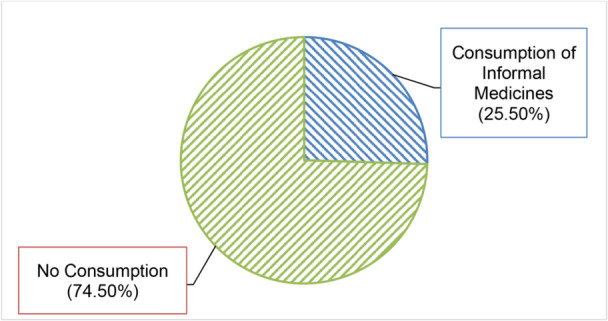
Prevalence of informally sold medicines consumption among the respondents.

Figure 2 presents the prevalence of informally sold medicines consumption among the respondents. The data indicate that 25.5% of individuals (*n* = 202; 95% CI: 22.5%–28.5%) use informal medicines, whereas 74.5% (*n* = 590) do not.

Table 2 provides insights into the patterns of consumption of informally sold medicines among the participants. Regarding the frequency of medicine purchases, 43.4% of respondents made two purchases per month, 33.6% made between two and four purchases, 16.5% made between four and six purchases, and 6.5% made six or more purchases. The results also revealed a pattern of multiple‐source medicine procurement. Medicines were predominantly purchased from retail pharmacies (83.8%), followed by endogenous traditional medicine centers (35.9%), informal markets (25.5%), public health facilities (22.8%), private health facilities (17.8%), and faith‐based health centers (3.4%). The main ailments for which informally sold medicines were consumed included malaria (78.5%), various ailments associated with pain (41.4%), arterial hypertension (15.3%), and Ear, Nose, and Throat (ENT) disorders (11.6%). 79.8% of the respondents practiced self‐medication, while 20.2% did not. Among the respondents, 58.1% had sought medical consultation at least once in the last 3 months before medicine purchases, whereas 41.9% had not. When adhering to medical prescriptions, 34% followed them fully, and 24.1% partially. Medicines prescribed were available in pharmacies regularly for 47.1% of respondents and irregularly for 52.9%. Awareness of the risks associated with informally sold medicines consumption was present in 53.8% of respondents, while 46.2% were unaware.

**Table 2:** Factors related to the consumption of informally sold medicines.

Variable	Count (792)	Frequency (%)
Medicine purchases per month		
2	344	43.4
[2–4]	266	33.6
[4–6]	131	16.5
≥ 6	51	6.5
Medicine purchase places		
Endogenous traditional medicine center	284	35.9
Pharmacies	664	83.8
Informal market	202	25.5
Public health center	180	22.8
Private health center	141	17.8
Faith‐based health center	27	3.41
Main ailments		
Malaria	622	78.5
Various ailments associated with pain	328	41.4
Arterial hypertension	121	15.3
Ear, Nose, and Throat (ENT) disorders	92	11.6
Diabetes	29	3.7
Digestive tract infections	29	3.7
Peptic ulcer disease	25	3.2
No affections	19	2.4
Hemorrhoids	8	1
Eye disorders	5	0.6
Sickle cell disease	4	0.5
Parasitosis	2	0.3
Typhoid fever	1	0.1
Urinary tract infections	1	0.1
Practices of self‐medication		
Yes	632	79.8
No	160	20.2
Medical consultation		
Yes	460	58.1
No	332	41.9
Adherence to medical prescriptions		
Complete	269	34
Partial	191	24.1
Awareness of risks associated with the consumption of informally sold medicines		
Yes	426	53.8
No	366	46.2
Availability of medicines in pharmacies		
Regular	373	47.1
Irregular	419	52.9

### 3.3 Predictors of the Consumption of Informally Sold Medicines

Table 3 summarizes the univariate, initial multivariate, and final multivariate logistic regression results, highlighting the factors significantly associated with the consumption of informally sold medicines in Cotonou.

**Table 3:** Logistic regression results on the determinants of the consumption of informally sold medicines.

	Univariate Model (*α* < 0.2)			Initial multivariate model (*α* < 0.05)		Final multivariate model (*α* < 0.05)		
Variables	PR	95% CI	*p* value	Adjusted PR	*p* value	Adjusted PR	95% CI	*p* value
Demographic								
Age	1.00	[0.99–1.01]	0.82					
Gender			0.98					
Male	1.00	—	**—**	**—**	**—**	**—**	**—**	**—**
Female	1.01	[0.73–1.40]	**—**	**—**	**—**	**—**	**—**	**—**
Household size			**0.17**					
< 4	1.00	—	**—**	1.00	—	**—**	**—**	**—**
≥ 4	1.30	[0.89–1.91]	**—**	0.71	0.30	**—**	**—**	**—**
Professional situation			**0.04**					
Employee (civil servant or private)	1.00	—	**—**	1.00	—	**—**	**—**	**—**
Self‐employed	1.78	[1.13–2.79]	**—**	1.63	0.18	**—**	**—**	**—**
Unemployed	1.15	[0.63–2.09]	**—**	1.04	0.94	**—**	**—**	**—**
Marital status			**0.12**					
Single	1.00	—		1.00	—			
Married	1.71	[0.99–2.93]	**—**	2.32	0.10	**—**	**—**	**—**
Divorced	2.24	[1.06–4.71]	**—**	1.85	0.39	**—**	**—**	**—**
Widowed	2.42	[0.74–7.92]	**—**	1.31	0.79	**—**	**—**	**—**
Socioeconomic								
Wealth quintiles			0.49					
Average	1.00	—		**—**	**—**	**—**	**—**	**—**
Poor	0.89	[0.60–1.32]		**—**	**—**	**—**	**—**	**—**
Very poor	1.02	[0.63–1.66]		**—**	**—**	**—**	**—**	**—**
Rich	0.77	[0.40–1.49]		**—**	**—**	**—**	**—**	**—**
Very rich	0.33	[0.07–1.51]		**—**	**—**	**—**	**—**	**—**
University education level			**0.002** **					
Yes	1.00	—	**—**	1.00	—	**—**	**—**	**—**
No	2.01	[1.26–3.22]	**—**	0.82	0.65			
Medicine use related								
Medicine purchases per month			**0.02** *					
Less than 2	1.00	—	**—**	1.00	—	1,00	—	—
2	1.22	[0.78–1.90]	**—**	1.48	0.28	0,96	[0.56–1.64]	0,89
3	1.49	[0.91–2.45]	**—**	1.46	0.35	1,31	[0.72–2.33]	0,37
4 and more	1.91	[1.27–2.85]	**—**	3.02	**0.002** **	1,90	[1.16–3.10]	**0.01** **
Medicine purchase places			**0.001** ***					
Pharmacy	1.00	—	**—**	1.00	—	1,00	—	**—**
Other sources	2.65	[1.79–3.93]	**—**	6.15	**0.001** ***	15.21	[7.52–30.78]	**0.001** ***
Main ailments			**0.02** *					
Less frequent (Others)	1.00	—		1.00	—	1,00	—	**—**
Frequent (malaria and pains)	1.91	[1.12–3.26]		2.25	0.11	2.02	[1.04–3.94]	**0.04** *
Practices of self‐medication			**0.001** ***					
No	1.00	—		1.00	—	1,00	—	**—**
Yes	24.05	[7.58–76.30]		18.39	**0.001** ***	95.02	[25.57–353.15]	**0.001** ***
Adherence to medical prescriptions			**0.001** ***					
Complete	1.00	—		1.00	—	**—**	**—**	**—**
Partial	2.46	[1.60–3.78]		1.51	0.12	**—**	**—**	**—**
Awareness of risks associated with the consumption of informally sold medicines			**0.001** ***					
Yes	1.00	—		1.00	—	1,00	—	**—**
No	2.82	[2.02–3.94]		2.83	**0.001** ***	2.31	[1.57–3.40]	**0.001** ***
Availability of medicines in pharmacies			**0.001** ***					
Regular	1.00	—		1.00	—	1,00	—	**—**
Irregular	3.56	[2.49–5.08]		3.84	**0.001** ***	3.42	[2.26–5.16]	**0.001** ***

*Note:* Univariate model = all variables; initial multivariate model = all variables with a significance level of *p* < 0.20 in the univariate model.

Final multivariate model = all variables with a significance level of *p* < 0.05 in the multivariate initial model.

Abbreviations: CI = confidence interval, PR = prevalence ratio.

*** *p* ≤ 0.001.

** 0.001 < *p* ≤ 0.01.

* 0.01 < *p* < 0.05.

The univariate logistic regression analysis revealed that demographic variables such as household size, professional situations, and marital status had significance levels of *p* = 0.17, *p* = 0.04 and *p* = 0.12 (*p* < 0.20) respectively, indicating their relevance for further analysis. Socioeconomic and medicine use‐related variables showed varying levels of significance. Specifically, university education levels, medicine purchases per month, medicine purchase places, main ailments, practices of self‐medication, adherence to medical prescriptions, awareness of risks, and availability of medicines in pharmacies all had significance levels, with several factors reaching highly significant levels (*p* < 0.02). Specifically, university education levels, medicine purchase places, practices of self‐medication, adherence to medical prescriptions, awareness of risks, and availability of medicines in pharmacies all had *p* < 0.001, highlighting their strong association with the consumption of informally sold medicines.

The initial multivariate logistic regression analysis from these variables selected revealed that only medicine use‐related variables such as medicine purchases per month, medicine purchase places, main ailments, practices of self‐medication, awareness of risks, and availability of medicines in pharmacies had significance association with the consumption of informally sold medicines. They were then retained for the final model.

### 3.4 Factors Associated With Informally Sold Medicines Consumption

Analysis of Table 3 reveals a very highly significant association between awareness of the risks linked to informally sold medicines and consumption of informally sold medicines. Individuals who were not aware of the risks consumed 2.31 times more informally sold medicines than those who were aware (adjusted PR = 2.31; 95% CI: 1.57–3.40 and *p* < 0.001). Also, a very highly significant association was noted between the habit of purchasing medicines and the consumption of informally sold medicines. Individuals who had other sources of supply, consumed 15.21 times the informally sold medicines compared to those who only obtained their supplies from pharmacies (adjusted PR = 15.21; 95% CI: 7.52–30.78 and *p* < 0.001). Likewise, a very highly significant association was noted between the availability of informally sold medicines in pharmacies and the consumption of informally sold medicines. The irregular availability of medicines in pharmacies justified an informally sold medicine consumption of 3.42 times compared to regular availability (adjusted PR = 3.42; 95% CI: 2.26–5.16 and *p* < 0.001).

Between the practice of self‐medication and the consumption of informally sold medicines, a very highly significant association was noted. Individuals who practiced self‐medication consumed 95.02 times more informally sold medicines than those who did not self‐medicate (adjusted PR = 95.02; 95% CI: 25.57–353.15 and *p* < 0.001).

At the same time, a highly significant association was noted between the frequency of drug purchases and informally sold medicines consumption. Individuals who purchased medications at least four times per month, consumed 1.90 times more informally sold medicines than those who purchased less than twice (adjusted PR = 1.90; 95% CI: 1.16–3.10 and *p* = 0.01).

Finally, between the main ailments and the consumption of informally sold medicines, we found a significant association. Individuals who were prone to the most common ailments (malaria or pains) consumed 2.02 times more informally sold medicines than those who did not (PR = 2.02; 95% CI: 1.04–3.94 and *p* = 0.04).

## 4 Discussion

### 4.1 Prevalence and Patterns of the Use of Informally Sold Medicines

The aim of this study was to determine the prevalence and factors associated with the use of informally sold medicines in Benin.

In the present study, the prevalence of informally sold medicines consumption was 25.5% (95% CI: 22.5%–28.5%). This prevalence is lower than 35.6% found by Abdoulaye et al. [11] in Cotonou (Benin) in 2006, and 72% by Angbo‐Effi et al. [16] in Abidjan (Ivory Coast) in 2011. The reduced proportion observed in this study partly reflects the significant increase in health infrastructure in Cotonou between 2006 and 2018, enhancing access to medical care. Additionally, since 2007, the Ministry of Health of Benin has intensified efforts to combat informally sold medicines through awareness campaigns on associated risks and strict enforcement against traffickers. These efforts have led to restricted access to informally sold medicines [4]. However, the still non‐negligible prevalence can also be explained by the existence of residual traffickers, particularly in homes and hidden corners of certain markets, which continue to facilitate the purchase of these informally sold medicines.

In the present study, more than half of the households purchased medicines at least twice during the month, with the pharmacy being the main source of supply (83.84%). It's therefore noted an acceptable use of pharmacies. These results are a far cry from those obtained in Qatar, which reported a nonsignificant difference between medicines purchasing locations [17]. The practice of self‐medication in the households surveyed was estimated at 79.8% with use of health services estimated at 58.1%. This rate of self‐medication practice is higher than that of 44% in 2006 [11]. A study carried out in Cotonou in 2016 on the acceptability and usefulness of rapid diagnostic tests in pharmacies in the management of uncomplicated malaria in adults found a self‐medication practice estimated at 74.5%, thus signifying an increase in the practice of self‐medication over the years in Cotonou [18]. The most common illnesses requiring medicines observed in households included malaria and various ailments associated with pain. This can be explained by the fact that Malaria is the most common cause of admission in hospital in Benin [4].

### 4.2 Predictors of the Consumption of Informally Sold Medicines

Among all demographic variables, age and gender were not associated with the informally sold medicine consumption, so they were not retained as predictors in the final analysis. These results are similar to those of Abdoulaye et al. [11], who found consumption of informally sold medicines without any significant difference in the proportions between young and older individuals, or between men and women, in Cotonou. These findings differ from those of Angbo‐Effi et al. [16] in Abidjan (Ivory Coast), where young individuals showed a higher prevalence of informal medicine consumption, with men consuming such medicines twice as often as women. This variation may stem from socio‐cultural differences between the two regions, as cultural and social factors can significantly influence reliance on the informal medicine market [19].

Regarding professional status, civil servants and private sector employees consumed fewer informally sold medicines compared to other professional groups. These could be explained by a good level of education, which leads to greater awareness of the dangers associated with consuming these medicines, thereby reducing the risk of using informally sold medicines [16]. This hypothesis is supported by the relationship between education level and the consumption of informally sold medicines in this study. The univariate regression analysis revealed that households with lower levels of education consumed more informally sold medicines (PR = 2.01; 95% CI: 1.26–3.22 and *p* = 0.002). This finding is also consistent with Apetoh et al. [4], who observed that individuals with lower education levels were more likely to purchase medicines from informal sources. However, these associations with the consumption of informally sold medicines were not statistically significant in our final multivariate model.

In our study, wealth quintile was not retained for the initial multivariate logistic regression analysis while household size was retained (*p* = 0.17), but its association with the consumption of informally sold medicines was not statistically significant in the final multivariate model. While larger households may experience greater financial strain, our findings did not confirm household size or self‐reported economic status as significant predictors. This contrasts with the findings of Abdoulaye et al. [11], who observed higher consumption rates in large, often polygamous households, and Angbo‐Effi et al. [16], who reported increased informal medicine use among low‐income households. However, Apetoh et al. [4] emphasized that informal medicine use is influenced by multiple factors beyond just financial limitations.

When associating all variables, the multivariate analysis revealed that only variables related to medicines were strongly associated with informally sold medicines consumption.

For the frequency of medicine purchases, the present study revealed that the prevalence of informally sold medicines consumption was higher among households that had a high frequency of medicine purchases per month (adjusted PR = 1.90; 95% CI: 1.16–3.10; *p* = 0.01). The higher the frequency of purchase is, the higher the medication needs will be. Therefore, the high prevalence of informally sold medicines consumption could be attributed to the unavailability of medicines at authorized facilities or to the proximity of informal sellers. According to Apetoh et al. [4], informal vendors of medicines are usually part of the neighborhood of inhabitants in Cotonou. The findings of this study also revealed that informally sold medicines consumption was very highly associated with supply outside pharmacies when adjusted for other factors (adjusted PR = 15.21; 95% CI: 7.52–30.78 and *p* < 0.001). Moreover, households who consumed informally sold medicines mentioned 3.42 times more irregular availability of medicines in pharmacies (adjusted PR = 3.42; 95% CI: 2.26–5.16 and *p* < 0.001). This refers to situations where medicines were either frequently out of stock, unavailable during certain times, or only available intermittently. This factor was very highly associated with the consumption of informally sold medicines when adjusted for other factors and poses the problem of stock shortages in pharmacies. This irregular availability led them to explore other sources, such as informal markets of medicines. Mengesha et al. [2], found in Ethiopia that barriers to accessing in‐demand essential medicines led to the emergence of substandard and falsified medicines. Likewise, the consumption of informally sold medicines was very highly associated with the practice of self‐medication, adjusted for other associated factors. Its prevalence in households that practiced self‐medication was 95.02 times that of other households (adjusted PR = 95.02; 95% CI: 25.57–353.15; *p* < 0.001). These findings reinforce the need for targeted interventions to regulate self‐medication and improve public awareness of its potential risks. Households that were prone to the most common ailments such as malaria or various pains consumed more informally sold medicines than others (adjusted PR = 2.02; 95% CI: 1.04–3.94; *p* = 0.04). This factor was significantly associated with informally sold medicines consumption in the present study, adjusted for other factors. Malaria is the most frequent ailments encountered in consultations in Benin health services [20].

As for awareness of the risks linked to the consumption of informally sold medicines, this factor remained highly associated with their use after adjustment for other variables in our study (adjusted PR = 2.31; 95% CI: 1.57–3.40; *p* < 0.001). Specifically, individuals who were unaware of the risks consumed informally sold medicines 2.31 times more than those who were aware of these risks. Hence, there is a need to inform the population, particularly, about the risks linked to the use of informally sold medicines based on concrete examples.

## 5 Conclusion

The study found that the prevalence of informally sold medicine consumption was notable among the population studied. This behavior was associated with several factors. These included limited awareness of the risks associated with informal medicines, exposure to malaria or various ailments associated with pain, self‐medication, high frequency of medicine purchases, irregular availability of medicines in pharmacies, and infrequent purchases in pharmacies. The findings suggest that the use of informally sold medicines is driven by both behavioral patterns and systemic limitations. This study highlights the need for a strategic fight against medicines sold informally, targeting consumers in particular. To reduce the associated risks and improve public health, it is crucial to strengthen consumer education, improve the availability of medicines and accessibility of medicines in the legal supply chain, and take targeted action against informally sold medicines.

## Author Contributions

**A. Narcisse D. Agbanou:** methodology, investigation, conceptualization, writing – original draft, writing – review and editing, data curation, software, formal analysis, resources, validation, visualization, and funding acquisition. **Fernand A. Gbaguidi:** supervision, validation, visualization, conceptualization, methodology, writing – review and editing, resources, project administration, and funding acquisition. **Fidèle Zinsou:** methodology, conceptualization, software, data curation, formal analysis, writing – review and editing, validation, and visualization. **Yossounon Chabi:** visualization, validation, writing – review and editing. **Habib Ganfon:** visualization, validation, writing – review and editing.

## Consent

All authors have read and approved the final version of the manuscript. A. Narcisse Degnon AGBANOU had full access to all of the data in this study and takes complete responsibility for the integrity of the data and the accuracy of the data analysis.

## Conflicts of Interest

The authors declare no conflicts of interest.

## Transparency Statement

The lead author, A. Narcisse D. Agbanou, affirms that this manuscript is an honest, accurate, and transparent account of the study being reported, that no important aspects of the study have been omitted; and that any discrepancies from the study as planned (and, if relevant, registered) have been explained.

## Data Availability

The data that support the findings of this study are available from the corresponding author upon reasonable request.

## References

[1] J. A. Nyarko , K. O. Akuoko , J. M. Dapaah , and M. Gyapong , “Exploring the Operations of Itinerant Medicine Sellers Within Urban Bus Terminals in Kumasi, Ghana,” Health Policy Open 5 (2023): 100108, 10.1016/j.hpopen.2023.100108. 38059004 PMC10696389

[2] A. Mengesha , H. Bastiaens , R. Ravinetto , L. Gibson , and R. Dingwall , “Substandard and Falsified Medicines in African Pharmaceutical Markets: A Case Study From Ethiopia,” Social Science & Medicine 349 (2024): 116882, 10.1016/j.socscimed.2024.116882. 38669893

[3] World Health Organization (WHO) , “Plus De Cent Tonnes De Faux Médicaments Saisis Et Cent Onze Faussaires Arrêtés Et Présentés Au Procureur: Le Ministre De L'intérieur Et La Sécurité Publique A Lancé Le Processus De Destruction Des Médicaments Frelatés [Internet],” WHO Regional Office for Africa, published 2017, https://www.afro.who.int/fr/news/plus-de-cent-tonnes-de-faux-medicaments-saisis-et-cent-onze-faussaires-arretes-et-presentes-au.

[4] E. Apetoh , M. Tilly , C. Baxerres , and J. Y. Le Hesran , “Home Treatment and Use of Informal Market of Pharmaceutical Drugs for the Management of Paediatric Malaria in Cotonou, Benin,” Malaria Journal 17 (2018): 354, 10.1186/s12936-018-2504-1. 30305107 PMC6180418

[5] A. Ouattara , “Achat De Médicaments De La Rue En Afrique: Essai De Compréhension D'un Comportement ‘Irrationnel’,” Market Management 9 (2011): 59–73.

[6] R. Pathak , V. Gaur , H. Sankrityayan , and J. Gogtay , “Tackling Counterfeit Drugs: The Challenges and Possibilities,” Pharmaceutical Medicine 37 (2023): 281–290, 10.1007/s40290-023-00468-w. 37188891 PMC10184969

[7] B. M. Ncube , A. Dube , and K. Ward , “Establishment of the African Medicines Agency: Progress, Challenges and Regulatory Readiness,” Journal of Pharmaceutical Policy and Practice 14 (2021): 29, 10.1186/s40545-020-00281-9. 33685518 PMC7938385

[8] S. Ozawa , D. R. Evans , S. Bessias , et al., “Prevalence and Estimated Economic Burden of Substandard and Falsified Medicines in Low‐ and Middle‐Income Countries: A Systematic Review and Meta‐Analysis,” JAMA Network Open 1 (2018): e181662, 10.1001/jamanetworkopen.2018.1662. 30646106 PMC6324280

[9] Y. H. Wada , A. Abdulrahman , M. Ibrahim Muhammad , V. C. Owanta , P. U. Chimelumeze , and G. M. Khalid , “Falsified and Substandard Medicines Trafficking: A Wakeup Call for the African Continent,” Public Health in Practice (Oxford, England) 3 (2022): 100240, 10.1016/j.puhip.2022.100240. 36101748 PMC9461548

[10] K. Karunamoorthi , “The Counterfeit Anti‐Malarial Is a Crime Against Humanity: A Systematic Review of the Scientific Evidence,” Malaria Journal 13 (2014): 209, 10.1186/1475-2875-13-209. 24888370 PMC4064812

[11] I. Abdoulaye , H. Chastanier , A. Azondekon , A. Dansou , and C. Bruneton , “Survey on the Illicit Drug Market in Cotonou, Benin in March 2003,” Medecine Tropicale: Revue Du Corps De Sante Colonial 66 (2006): 573–576. 17286024

[12] Benin Agency for Medicines and Health Products (ABMed), “Répertoire Des Établissements Pharmaceutiques [Internet],” Cotonou: ABMed, published December 23, 2024, https://abmed.bj/referentiels-listes/repertoire-des-etablissements-pharmaceutiques-liste-agrements.

[13] National Institute of Statistics and Demography (INStaD), “Effectifs De La Population Des Villages Et Quartiers De Ville du Bénin (RGPH‐4, 2013) [Internet],” INStaD, published 2016, https://instad.bj/images/docs/insae-statistiques/demographiques/population/Effectifs%20de%20la%20population%20des%20villages%20et%20quartiers%20de%20ville%20du%20benin/Cahier%20Village%20RGPH4%202013.pdf.

[14] Benin Agency for Medicines and Health Products (ABMed), “Bulletin D'information De Vigilance Des Produits De Santé [Internet],” Cotonou: ABMed, published 2023, https://abmed.bj/assets/img/pages/Bulletin-info-vigilance-PS_ed-01.pdf.

[15] USAID Global Health Supply Chain Program, “Benin Destroys 118 Tons of Counterfeit Pharmaceuticals and Develops In‐Country Capacity in Pharmaceutical Waste Management [Internet],” published 2018, https://www.ghsupplychain.org/news/benin-destroys-118-tons-counterfeit-pharmaceuticals-and-develops-country-capacity-0.

[16] K. O. Angbo‐Effi , D. P. Kouassi , G. H. A. Yao , A. Douba , R. Secki , and A. Kadjo , “Facteurs Déterminant La Consommation Des Médicaments De La Rue En Milieu Urbain,” Santé Publique 23 (2011): 455–464, 10.3917/spub.116.0455. 22365044

[17] A. A. Alfadl , M. I. M. Ibrahim , F. A. Maraghi , and K. S. Mohammad , “General Public and Community Pharmacists Perception on Counterfeit Medicines: A Preliminary Cross‐Sectional Study in Qatar,” Journal of Clinical and Diagnostic Research 12, no. 01 (2018): IC01–IC06, 10.7860/JCDR/2018/28451.11136. PMC5071965 27790465

[18] M. F. Hounnamon , “Acceptabilité Et Utilité Des Tests De Diagnostic Rapide En Pharmacie Dans La Prise En Charge Du Paludisme Simple Chez L'Adulte: Étude Pilote Réalisée à Cotonou” (Pharm thesis, Faculty of Health Sciences, University of Abomey‐Calavi, 2016).

[19] A. Dagrou and V. Chimhutu , “I Buy Medicines From the Streets Because I Am Poor: A Qualitative Account on Why the Informal Market for Medicines Thrive in Ivory Coast,” Inquiry: A Journal of Medical Care Organization, Provision and Financing 59 (2022): 469580221086585, 10.1177/00469580221086585. 35311389 PMC8941685

[20] Ministry of Health of Benin. “Annuaire Des Statistiques Sanitaires Année 2019.” Cotonou: Ministry of Health, 2020, 244.

